# TGF-β in T Cell Biology: Implications for Cancer Immunotherapy

**DOI:** 10.3390/cancers10060194

**Published:** 2018-06-11

**Authors:** Amina Dahmani, Jean-Sébastien Delisle

**Affiliations:** 1 Centre de Recherche de L’hôpital Maisonneuve-Rosemont, 5415 Boul. de L’Assomption, Montréal, QC H1T 2M4, Canada; amina.dahmani1@gmail.com; 2 Hematology-Oncology service, Hôpital Maisonneuve-Rosemont, Department of Medicine, Université de Montréal, Montréal, QC H1T 2M4, Canada

**Keywords:** TGF-β, T cells, cancer, immunotherapy

## Abstract

Transforming Growth Factor beta (TGF-β) is a pleiotropic cytokine produced in large amounts within cancer microenvironments that will ultimately promote neoplastic progression, notably by suppressing the host’s T-cell immunosurveillance. This effect is mostly due to the well-known inhibitory effect of TGF-β on T cell proliferation, activation, and effector functions. Moreover, TGF-β subverts T cell immunity by favoring regulatory T-cell differentiation, further reinforcing immunosuppression within tumor microenvironments. These findings stimulated the development of many strategies to block TGF-β or its signaling pathways, either as monotherapy or in combination with other therapies, to restore anti-cancer immunity. Paradoxically, recent studies provided evidence that TGF-β can also promote differentiation of certain inflammatory populations of T cells, such as Th17, Th9, and resident-memory T cells (Trm), which have been associated with improved tumor control in several models. Here, we review current advances in our understanding of the many roles of TGF-β in T cell biology in the context of tumor immunity and discuss the possibility to manipulate TGF-β signaling to improve cancer immunotherapy.

## 1. Introduction

Transforming growth factor beta (TGF-β) is a major determinant of hematopoietic and immune cell development and physiology. The objective of this review is to provide an overview of TGF-β biology in conventional T cells, specifically in relation to cancer biology and immunotherapy. TGF-β is an evolutionally conserved cytokine that belongs to a large family of morphogens and growth factors [1]. In mammals, TGF-β is widely expressed and modulates a large spectrum of biological processes including normal development, carcinogenesis, and immune responses. This great versatility and pleiotropy requires input from a multitude of other pathways (reviewed in [2,3,4,5,6,7,8,9]). The roles of TGF-β in T cells and cancer immunology remain likewise highly context-dependent. 

The development and progression of cancer are also markedly impacted by the effects of TGF-β [10]. Along with numerous effects on the neoplastic cells and tumor stroma, the multipronged effects of TGF-β on immune cells shape the cancer microenvironment [11]. In cancer, TGF-β has been shown to support the evasion of cancer cells from immune surveillance and to contribute to the subversion of the immune system from being an extrinsic tumor suppressor to a promoter of malignant growth and spread [12,13,14,15,16]. Hence, in addition to the strong rationale to use TGF-β antagonist therapy to directly target cancer cells and tumor stroma, the prospect of reinvigorating anti-tumor immunity with TGF-β inhibition is appealing, especially in an emerging era of cancer immunotherapy [17]. However, TGF-β is more than an immunosuppressive cytokine and can also promote the differentiation, function, and homeostasis of certain inflammatory populations of T cells, such as T-helper 17 (Th17), Th9, and resident memory T cells (Trm) [18]. It is to be anticipated that the current and future therapies that will most efficiently target TGF-β in cancer will benefit from the knowledge accumulated over more than two decades on the role of TGF-β in T-cell biology. In mammals, three TGF-β isoforms have been identified: TGF-β1, TGF-β2, and TGF-β3. In the immune system, TGF-β1 isoform is predominant and controls the development, homeostasis, differentiation, and function of multiple immune cell types [18,19]. The central role of TGF-β as a regulator of the immune system was revealed by TGF-β1-deficient mice that develop a severe multifocal and fatal inflammatory response, associated with disruption of different immune cell compartments including T cells, B cells, macrophages, and dendritic cells [20,21,22,23]. This lethal inflammatory disorder was also observed in mice with T-cell-specific overexpression of a dominant-negative TGFbRII (dnTGFbRII) [24], or deletion of either TGFbRI or TGFbRII [25,26,27] and attenuated in T-cell-deficient mice [28]. Importantly, these studies confirmed that the essential role of TGF-β in self-tolerance hinged on T cells, and that multiple mechanisms were in play. Notably, conventional T cells underwent uncontrolled proliferation, activation, and effector differentiation while regulatory T cells (Tregs), which are essential for peripheral tolerance [29], were reduced. These studies established TGF-β as both a modulator of T-cell physiology and differentiation. 

## 2. TGF-β Secretion, Activation and Signaling

TGF-β is synthesized by several cell types, including most hematopoietic cell subtypes, as an inactive molecule, composed of a homodimer of mature TGF-β non-covalently associated with the latency-associated protein (LAP). This small latent complex is either secreted or associated with another protein, latent-TGF-β-binding protein (LTBP), that directs TGF-β to the extracellular matrix for future activation [19,30]. To mediate its biological functions, mature TGF-β must be released from LAP. This action can be achieved through several mechanisms including dissociation by acidic pH, interaction with integrins, or proteolysis of LAP by matrix metalloproteinases. In physiological and pathological conditions, integrins play a crucial role in the activation of TGF-β in the immune system. Specifically, the integrins αvβ6 and αvβ8 are essential to the regulation of immune homeostasis, as mice lacking both functional αvβ6 and αvβ8 integrins reproduced the phenotype seen in TGF-β1^−/−^ mice [31]. Conditional deletion of integrin αvβ8 on leukocytes causes severe inflammatory bowel disease and age-related autoimmunity in mice, suggesting a key role of αvβ8 integrin-mediated TGF-β activation by leukocytes in maintaining T-cell homeostasis and controlling inflammation [32]. This autoimmune phenotype was largely due to lack of αvβ8 on dendritic cells, as mice lacking αvβ8 principally on dendritic cells develop similar phenotype to mice lacking αvβ8 on all leukocytes. In contrast, mice lacking αvβ8 on T cells alone are phenotypically normal [32]. These results were further reinforced by the finding that a specific loss of integrin αvβ8 in a specialized subset of CD103^+^dendritic cells in the intestine abrogated their ability to induce Tregs [33]. Moreover, it has been shown that integrin αvβ8 expression on dendritic cells plays a critical role in the differentiation of Th17 cells. Mice lacking integrin αvβ8 in dendritic cells have reduced numbers of Th17 cells in the colonic mucosa and fail to generate highly pathogenic Th17 cells during experimental autoimmune encephalomyelitis [34]. In addition to dendritic cells, Tregs can capture latent TGF-β at their surface and activate it. This capture involves latent TGF-β binding to a transmembrane protein called glycoprotein A repetitions predominant, GARP [35,36,37,38]. Moreover, the gene encoding β8 subunit of the integrin αvβ8 has been shown to be selectively expressed in both mouse and human Tregs, but not conventional T cells [39,40].These studies showed that mouse Tregs require integrin αvβ8 to activate TGF-β1, and that αvβ8-deficient Tregs are unable to induce differentiation of naive T cells into IL-17 expressing Th cells (Th17) in vitro. Finally, αvβ8 integrin is constitutively expressed on thymic Treg and mediates the release of active TGF-β1 from the latent TGF-β1/GARP complex.

Once released, active TGF-β binds to dimeric TGFb type 2 receptor (TGFbRII), a serine/threonine kinase that recruits and activates a second dimeric type 1 receptor (TGFbRI) to form a tetrameric receptor complex that initiates signaling pathways through its kinase activity. Activated TGFbRI phosphorylates the mothers against decapentaplegic homolog (SMAD) 2 and SMAD3, which are transcription factors that subsequently form a complex with SMAD4 or the transcriptional intermediary factor 1 gamma (TIF1γ) [41,42]. This complex translocates into the nucleus, where it recruits transcription cofactors to modulate the expression of target genes. In addition, activated TGF-β receptor complexes can also trigger SMAD-independent, non-canonical pathways, such as several mitogen-activating protein kinases (MAPKs) pathways, Rho-like GTPase signaling pathways, and phosphatidylinositol-3-kinase/AKT pathways, to regulate a wide array of functions in different cellular and tissues contexts [8]. This multiplicity of signaling pathways and the inherent plasticity of SMAD signaling downstream of TGF-β receptors mediated notably by the recruitment of co-factors and post-translational modifications, as well as the diverse mechanisms that control the bioavailability of TGF-β, contribute to the pleiotropic nature of TGF-β actions [1,9,41].

## 3. TGF-β: Suppressor of T-Cell Proliferation and Effector Functions

TGF-β is implicated in the regulation of diverse immune responses ranging from infections, suppression of autoimmune disorders, and cancer through direct regulatory effects on multiple immune cell types, including lymphocytes, dendritic cells, and numerous myeloid subsets [14,18]. In TGF-β1-deficient and in conditional T-cell specific TGFbR-deficient mice, T cells showed increased proliferation, activation, and T-helper 1 (Th1), as well as Th2 cytokine production [43,44]. Although invaluable, these mouse models did not allow one to fully dissect the role of TGF-β at different stages of development. The deletion of TGFbRII in mature T cells does not result in autoimmunity. Rather, it predisposes T cells to enhanced reactivity and expansion following stimulation or homeostatic expansion [45]. Likewise, the methods to abrogate TGF-β signaling (complete knock-out versus dominant negative receptor expression) can result is different outcomes, suggesting a dose-effect in TGF-β signaling [46]. In addition, abrogation of canonical TGF-β signaling intermediates such as the SMADs do not recapitulate the severe autoimmune features observed in TGFbR-deficient mice, implying a role for non-canonical TGF-β signaling pathways in the control of inflammation [47,48]. These relevant distinctions notwithstanding, common features are that TGF-β inhibits conventional T-cell proliferation and effector functions.

T-cell proliferation. It has been shown in several cell types that TGF-β is a potent inhibitor of cellular proliferation. In hematopoietic stem cells, TGF-β regulates quiescence through several mechanisms including modulation of mechanistic target of rapamycin (mTOR) and Forkhead box O3 (FOXO3] [49,50]. In turn, TGF-β signaling in stem cells was recently found to be dependent on Src homology region 2 domain-containing phosphatase-1 (SHP-1), which positively regulates TGF-β signaling [51]. In T cells, the main drivers of proliferation are cytokines, signals downstream of the T-cell receptor (TCR), and co-stimulatory molecules. In vitro, exogenous TGF-β suppresses IL-2-dependent proliferation of activated human T cells and partially inhibits IL-2 receptor expression [52]. This effect could only be partially reversed by the addition of exogenous IL-2, suggesting that the cytostatic effect of TGF-β on T cells is not only due to the suppression of IL-2 production. TGF-β was shown to inhibit IL-2 production through direct inhibition of IL-2 promoter activity in a SMAD3-dependant manner [53,54]. In vivo, T cell-specific expression of a dnTGFβRII expression has demonstrated that TGF-β negatively regulates CD4 and CD8 T cell expansion [24,44]. TGF-β can also inhibit T cell proliferation through the transcriptional regulation of cell cycle target genes, including cyclin-dependent kinases inhibitors (p15, p21, and p27, and c-myc) [55,56]. The mechanisms by which TGF-β interacts with these genes are still unclear, but the canonical TGF-β mediator SMAD3 has been shown to be a key mediator of the growth inhibitory effect of TGF-β in T cells [57]. T cells from SMAD3-null mice are resistant to the antiproliferative effect of TGF-β [58,59,60]. However, the anti-proliferative effects of SMAD3 may be more prominent in CD4^+^ than in CD8^+^ T cells [61], suggesting that SMAD2-dependent or SMAD-independent pathways limit CD8^+^ T-cell proliferation [46,62]. Another study suggests that the TGF-β/SMAD3 pathway restricts CD4^+^ T-cell growth and proliferation by mitigating the effects of CD28 costimulation, resulting in decreased mTOR signaling [63]. Likewise, TGF-β was also shown to negatively regulate NK cell functions by inhibiting mTOR signaling [64].

T-cell activation. In addition, TGF-β impedes T cell activation by interfering with proximal TCR signaling events such as phosphorylation and activation of the Tec kinase Itk, Ca^2+^ mobilization, NFATc translocation, and activation of the mitogen-activated protein kinase ERK Tec kinase, which are critical for T cell differentiation [65,66]. TGF-β also abrogates TCR-mediated signaling by upregulating tyrosine phosphatases such as SHP-1, which in turn negatively regulate molecules downstream of the TCR such as the protein tyrosine kinases (PTK) P56lck, P59fyn, and Zap-70 [67]. Of particular relevance to this report, T lymphocytes deficient for Diacylglycerol kinases DGK-ζ (known to play a role in TCR signal transduction by initiating degradation of the second messenger DAG) and engineered to express a cancer targeting chimeric antigen receptor (CAR) were less sensitive to TGF-β mediated suppression than their wild type counterparts [68]. The mechanistic underpinnings of such effect are unclear, but it was previously shown that mediators downstream of TCR signaling can actively suppress TGF-β signaling, thereby allowing T cells to escape TGF-β regulation during activation [69]. The interplay between TCR signaling and TGF-β involves several feedback loops with the suppressive effects of TGF-β being most important at the initiation of the response but not in post-activation, actively proliferating T cells [70].

**Figure 1:**
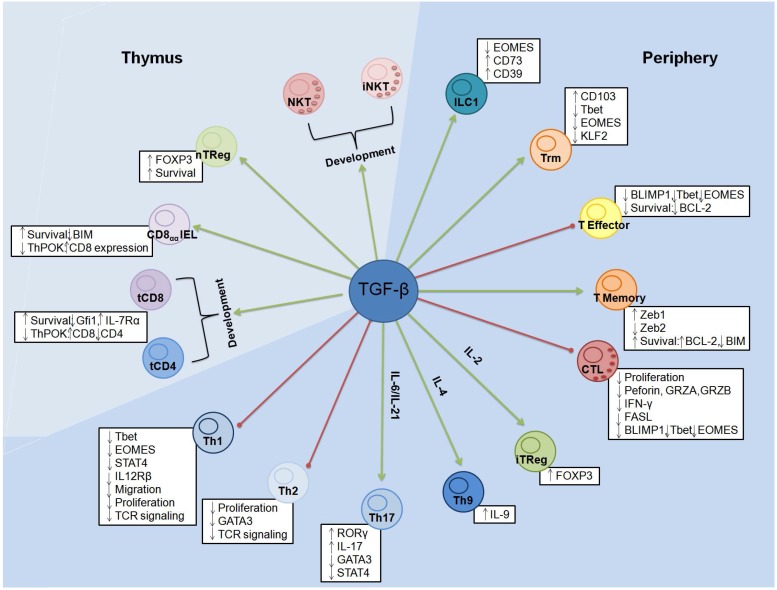
Overview of TGF-β effects on T-cell subsets. Graphical representation of positive (green) or inhibitory (red) effects of TGF-β signaling on T-cell differentiation across developing T cells (in the thymus, light blue) or mature T-cell subsets (in the periphery, dark blue). Mechanistic or physiologic impact of TGF-β signaling on the various T-cell subsets indicated in the white boxes).

T-cell effector functions. TGF-β has also been shown to be a potent suppressor of CD8^+^ cytotoxic T cell (CTL) effector functions through diverse mechanisms, including inhibition of perforin, Granzyme B and A, interferon-gamma (IFN-γ), and FAS ligand (FASL) expression (Figure 1). Thomas and Massagué showed that systemic neutralization of TGF-β in vivo results in tumour eradication, associated with an increase in CD8^+^ T-cell mediated tumour-cell-specific cytotoxicity [71]. Moreover, the genes encoding for the effectors molecules of CTL response, as well as their intracellular concentration, were downregulated in T cells activated in vitro in the presence of TGF-β. Indeed, TGF-β neutralization in vivo permitted to recover the expression of these molecules. Consistent with this, many other studies showed that the adoptive transfer of tumor-specific CTL engineered to be desensitized to TGF-β displayed enhanced CTL function and antitumor responses [72,73,74,75]. Central to the inhibition of cell cytotoxic mechanism by TGF-β is the regulation of T-cell activation-associated transcription factors expression. Notably, the master regulators of CTL differentiation and activation T-BET, EOMES, and BLIMP-1 are directly targeted by TGF-β. The transcription factors T-BET and EOMES promote the expression of type 1 cytotoxic molecules (e.g., granzyme B, perforin, and IFN-γ) [76,77,78,79,80]. In the B16 melanoma murine model, in vivo administration of ALK5 inhibitors (which block phosphorylation of receptor SMADs by occupying the ATP binding site of TβRI domain) suppressed tumor progression and enhanced CTL responses through the restoration of EOMES expression [81]. Likewise, TGF-β inhibits T-BET expression in Th1 cells and has been shown to inhibit the acquisition of effector functions in ex vivo-stimulated memory human CD8^+^ T cells reactive melanoma antigens [43,82]. The transcriptional repressor BLIMP-1 also promotes CTL effector differentiation and actively suppresses T-cell memory transcriptional program [83,84]. In mouse models of established tumors, Lin and al. showed that tumor-derived TGF-β directly suppresses CTL function by inhibiting BLIMP-1 expression through the stimulation of miR-23a expression [85]. The abrogation of miR-23a expression ameliorated TGF-β–induced CTL suppression and restored Granzyme B and IFN-γ expression, thereby mitigating TGF-β–induced immunosuppression. The inhibition of T-cell activation, proliferation, and cytotoxicity is intricately linked to T-cell differentiation mechanisms in which TGF-β intervenes at several levels to influence T-cell fates as described in the next section. 

## 4. TGF-β: Master Regulator of T-cell Homeostasis and Differentiation

T-cell development and homeostasis. From T-cell ontogeny to the regulation of T-cell survival and death, TGF-β modulates several aspects of T-cell fate decisions (recently reviewed in [18]) that are directly relevant to cancer immunobiology and immunotherapy (Figure 1). During T-cell development in the thymus, TGF-β acts as a pro-survival factor for developing conventional CD4^+^ and CD8^+^ T cells selected for low avidity against self-antigens through a positive effect on IL-7 receptor-alpha (IL-7Rα) expression [86]. Moreover, TGF-β not only promotes CD4^+^ and CD8^+^ T-cell survival but also the ontogeny of thymic regulatory (tTregs) and several subsets of innate T cells (invariant natural killer T cells-iNKT and CD8αα^+^TCRαβ^+^ intra-epithelial lymphocytes-IEL). In this setting, TGF-β may attenuate negative selection following strong interactions between the developing precursors and their antigenic ligand [87].

Post-thymic mature naïve CD4^+^ T cells continue to receive pro-survival signals from TGF-β in the periphery, which contributes to maintain a diverse T-cell repertoire [88]. The role of TGF-β in CD8^+^ T-cell homeostasis is more complex and highlights the pleiotropy of this cytokine. Importantly, it reemphasizes the importance of T-cell differentiation status on the outcome of TGF-β signaling. Indeed, several models have shown that TGF-β prevents T-cell activation and expansion [24,45] promotes survival of post-activation and memory T cells [89,90,91], but favors apoptosis of effector T cells [92]. Although most mechanisms that underlie these divergent influences are still unknown, it was recently shown that TGF-β upregulates *Zeb1* and inhibits *Zeb2*, which are transcription factors that respectively promote memory T-cell survival and favor terminal effector T-cell differentiation in murine lymphocytic choriomeningitis virus (LCMV) infection [93]. Interestingly, TGF-β modulation of *Zeb* transcription factors is a cardinal feature of epithelial-mesenchymal transition (EMT), which is at the origin of the metastatic behavior of cancer cells [94,95,96]. In the case of CD8^+^ T cells, conditional deletion of *Zeb1* led to low expression of the anti-apoptotic BCL-2 molecule relative to the pro-apoptotic molecule BIM in memory T cells. This is in contrast to the finding that low BCL-2 expression was proposed as a mechanism to explain the pro-apoptotic role of TGF-β in effector CD8^+^ T cells [92]. It is therefore likely that depending on different cellular contexts, TGF-β modulates opposing cellular fates through divergent modulation of the same pathways.

CD4^+^ T-cell differentiation. In order to mount effective immune responses, T cells must differentiate into specialized subtypes. Best described for CD4^+^ helper T cells [97], T-cell differentiation is heavily influenced by TGF-β (Figure 1). Consistent with a predominantly immunoregulatory role and of particular relevance to T-cell responses against cancer, TGF-β has been shown to significantly blunt Th1 and Th2 effector differentiation [24,43,98,99]. The CD4^+^ Th1 response, which overlaps with CTL differentiation in CD8^+^ T cells, is notably characterized by IFN-γ production and responses against virus-infected cells and cancers. Th1 responses are significantly inhibited by TGF-β, which suppresses the expression of the Th1 fate determining transcription factors T-BET, EOMES, and STAT4 [25,43,91,99]. In addition, TGF-β favors Treg differentiation from uncommitted peripheral CD4^+^ T cells through the induction of the Treg signature transcription factor FOXP3 [27,100,101,102,103]. Both thymus-derived and induced Tregs will suppress immune responses through several mechanisms, including the production and activation of TGF-β [104]. Along with the suppression of T-cell activation and cytotoxicity, the mitigation of Th1 responses and the induction of Treg differentiation are central to the immunoregulatory role of TGF-β in tumors [14]. The production of TGF-β by the tumor cells, immature dendritic cells, and stromal element favor the recruitment and in situ conversion of effector T cells into Tregs at least in part through the direct action of SMAD3 on the FOXP3 gene promoter [100,105,106,107,108].

Despite undisputable immunoregulatory effects, TGF-β also controls T-cell differentiation programs leading to inflammatory subset generation. Among TGF-β-dependent subsets, Th9, Th17, and CD8^+^ resident memory (Trm) T cells are of particular relevance to cancer (Figure 1). Whether Th17 contributes to pro- or anti-tumor inflammation remains controversial and context-dependent (reviewed in [109]). Importantly, TGF-β is one of the factors that may explain the dual effects of Th17 T cells in cancer. The role of TGF-β in Th17 fate determination is both direct and indirect. Along with IL-6, IL-1β, IL-23, and IL-21, TGF-β directly supports the expression of the Th17 lineage determining transcription factor RORγt in mouse CD4^+^ T cells (RORC in humans) [110]. Moreover, the inhibition of other differentiation programs (namely, Th1 and Th2) through TGF-β favors Th17 generation [111,112,113]. However, beyond the signals that initially trigger the Th17 program, several other cytokines can further specialize Th17 cells, or reverse their phenotype and function. Importantly, TGF-β itself alters the Th17 fate at several stages. In addition to the cytokine context that will favor Th17 instead of Treg differentiation, a determining and often underappreciated variable is the concentration of TGF-β. At high concentration, TGF-β favors Treg over Th17 differentiation through inhibition of IL-23R expression and direct antagonism of FOXP3 on RORγc expression [114]. In addition, the multiplicity of signaling pathways downstream of TGF-β receptors can also contribute to lineage determination. The TGF-β canonical mediator SMAD4 articulates Treg but not Th17 differentiation, which was shown to rely on non-canonical AKT and MAPK signaling [48,115,116,117]. Moreover, within the context of tumors, ongoing TGF-β signaling could boost several immunoregulatory properties of Th17 cells, among them, the suppression of T-BET and the expression of the ectonucleotidases CD73 and CD39 leading to adenosine production and suppression of immune responses [118,119]. Moreover, in pre-clinical models, a subset of Th17 induced by TGF-β and IL-6 and expressing high levels of aryl hydrocarbon receptor (AhR) was found to secrete IL-10 and have immunoregulatory properties (Treg17) [120,121]. These data infer that the optimal mobilization of Th17 for cancer therapy may require the generation of highly inflammatory Th17 without TGF-β, or the neutralization of high TGF-β concentration found in tumors [122].

The Th9 fate is characterized by the secretion of IL-9 by CD4^+^ T cells, leading to several pro-inflammatory and anti-cancer effects (reviewed in [123]). Th9 cells, close relative of Th2 T cells, are generated following TGF-β and IL-4 signaling leading to expression of the transcription factor PU.1 and IL-9 production. While controversy persists on whether a Th9 response is beneficial or harmful in human cancer [124], Th9 T cells have several properties that make them appealing as an anti-cancer subset. Th9 and IL-9 were shown to have direct pro-apoptotic effects on cancer cells [125] and pro-survival effects on T cells. Other effects include mast cell stimulation, IFN-γ production by T cells and NK cells, and their recruitment along with dendritic cells and other leukocytes [126,127]. Intriguingly, it was also shown in pre-clinical models that the anticancer effects following agonistic stimulation of glucocorticoid-induced TNFR-related protein (GITR) expressed on T cells occurred through the development of a Th9 response [128,129]. Whether Th9 immunity develops under treatment with other T-cell co-stimulation modulators such as immune checkpoint blockade in humans is being investigated and should provide important prognostic and mechanistic insights [130].

CD8^+^ T-cell differentiation: although most extensively described for CD4^+^ T cells, the effects of TGF-β on T-cell differentiation schemes extend to CD8^+^ T cells. The induction of FOXP3 in CD8^+^ T cells through TGF-β can lead to the differentiation CD8^+^ Tregs with suppressive functions in inflammatory disease and human cancer [131]. Likewise, IL-17-producing CD8^+^ T cells (Tc17) have been described in numerous settings, but the contribution of TGF-β to Tc17 skewing may not be as important as for Th17 differentiation given that the differentiation of Tc17 is not impaired in transgenic mice expressing dnTGFbRII [132]. Interestingly, the injection of CD8^+^ cultured in Th9 skewing conditions (IL-4, TGF-β, and anti-IFNγ) was shown to give rise to Tc9 with stronger anti-tumor tumor effects than IFN-γ cytolytic CD8^+^ T cells in pre-clinical cancer models [133]. Hence, the modulation of Treg, Th17, and Th9 fates in cancer is likely to have important impact on the entire conventional T-cell compartment.

A CD8^+^ T-cell subset with strong relevance to cancer is a group of T cells designated as resident-memory T cells (Trm). These long-lived T cells infiltrate tissues and are retained peripherally to mediate rapid responses to invading pathogens [134]. These T cells are diverse and vary according to their tissue of residence. TGF-β potently induces CD103, an integrin favoring direct contact with epithelia, and downregulates *KLF2*, a transcription factor favoring egress from secondary lymphoid organ. The downregulation of *KLF2* leads to decreased sphingosine-1-phosphate receptor 1 (S1P1) expression which establishes T-cell retention in tissues [135,136]. In addition, Trm differentiation has been show to rely on T-BET and EOMES downregulation, which can be mediated by TGF-β [137], as in the inhibition of Th1 differentiation. In several cancers, the presence of CD8^+^CD103^+^ infiltrating T cells within tumor microenvironment correlates with improved survival [138,139,140,141]. This raises the possibility that the most effective tumor-infiltrating lymphocytes (TILs) that are stimulated through immune checkpoint blockade [142,143,144] or harvested and re-infused as cell therapy [145,146] are partly attracted and retained within tumor environments through TGF-β-dependent mechanisms [139]. As described below, the role of TGF-β signaling in shaping the immune environment of cancer is far more complex, but enhancing tumor-residence of T cells may represent a promising approach to enhance current immunotherapies.

Innate T-cell differentiation. Finally, the role of innate lymphoid cell (ILC), innate-like T cells (ILTC), natural killer (NK) cells, and γδ T cells is increasingly studied in the context of cancer immunobiology. It has been known for a long time that TGF-β potently inhibits NK cell functions [147,148,149,150], and that it can induce γδ Tregs [151,152]. More recently, TGF-β has emerged as a key factor determining the differentiation and characteristics of tissue resident group 1 ILC (ILC1) [153]. Such unconventional T cells are cytotoxic and secrete IFN-γ, thereby mediating early responses against neoplastic cells in experimental models [154]. Whether TGF-β modulates the responses by resident innate cells is currently unknown. However, the differentiation of group 1 ILC (ILC1) from innate NKp46^+^ lymphoid precursors in mouse salivary glands has been shown by Cortez and colleagues to depend on the TGF-β-mediated suppression of the transcription factor EOMES through Jun N-terminal kinase-dependent signaling [153]. Importantly, TGF-β-imprinted phenotypic and functional features in salivary glands ILC1 that were not present in ILC1 from different organs. Several immunoregulatory characteristics such as low IFN-γ secretion and the expression of CD39 and CD73 were induced in a TGF-β-dependent manner specifically in the salivary gland ILC1 cells. Moreover, TGF-β has been found to differentiate NK cells into ILC1 within cancer microenvironment, resulting in decreased anti-tumor effects and escape from NK-mediated tumor control [155]. Hence, the impact of TGF-β on ILC1 differentiation and characteristics is highly context-dependent and remains to be fully defined in cancer.

Globally, the extensive work done on the roles of TGF-β in T-cell differentiation reveals that the impact of TGF-β depends on context (i.e., other signals and cellular states), timing of exposure, and concentration. Hence, the outcome of TGF-β signaling modulation in cancer therapy is likely to depend on these important variables.

## 5. TGF-β: Architect of the Immune Tumor Microenvironment and Therapeutic Opportunities

**Figure 2:**
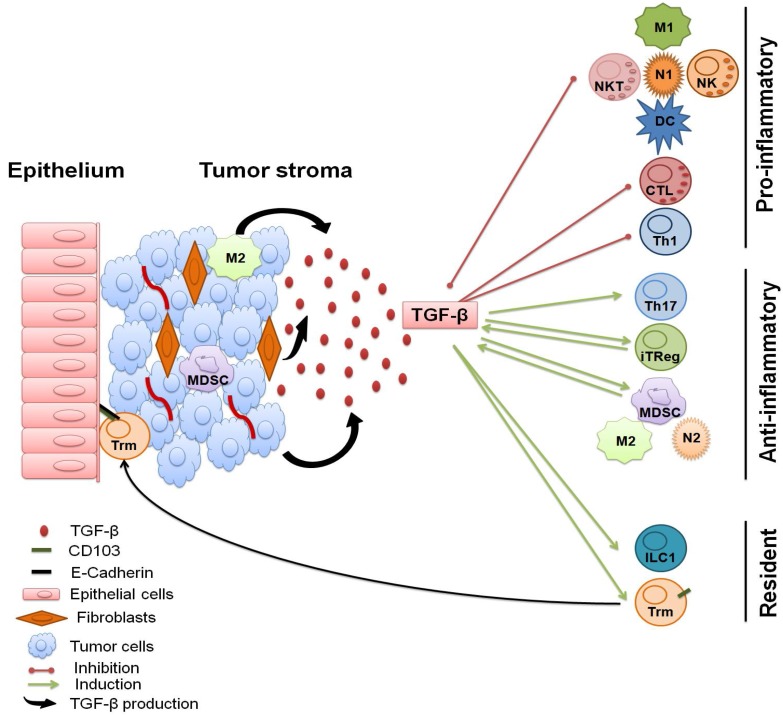
Schematic representation of TGF-β as a modulator of the tumor microenvironment. Representation of inflammatory lymphoid and myeloid (DC-dendritic cells, M1 inflammatory macrophages, or neutrophils—N1) immune cells was negatively regulated (red) by TGF-β, and anti-inflammatory subsets were promoted (green) by the actions of TGF-β (including myeloid-derived suppressor cells-MDSC, anti-inflammatory macrophages—M2 or neutrophils—N2). The action of TGF-β in the migration and retention of T cells is exemplified by the effect on Trm differentiation and can result in both tumor infiltrating lymphocyte (TIL) generation or lead to exclusion from tumors when TGF-β is produced by surrounding stromal cells.

The presence of TGF-β in cancer microenvironments impacts several biological processes that ultimately contribute to cancer progression. The importance of TGF-β signaling on cancer cells, or cancer cell resistance to TGF-β signaling during cancer progression, as well as the impact of TGF-β in non-immune and non T-cell subsets that compose the neoplastic microenvironment, has been reviewed elsewhere and will not be extensively discussed here [10,11,14,16,156,157,158,159,160]. It should nonetheless be mentioned that TGF-β from several sources can have a determining impact on T cells within the cancer microenvironment. This section will focus on TGF-β signaling as a direct and indirect modifier of conventional T-cell function in the cancer microenvironment (depicted in Figure 2) and how TGF-β signal modulation can be used therapeutically.

TGF-β, T cells, and the tumor microenvironment: The immune cell composition and context of human cancers has important biologic and clinical implications [161,162,163,164]. Globally, the presence of abundant TGF-β in cancer microenvironments adversely impacts cancer prognosis. Importantly, this is secondary to several TGF-β-dependent processes, such as angiogenesis, fibrosis, and EMT that extend beyond immunosuppression (reviewed in [165]). Perhaps best described for colo-rectal cancer, the co-occurrence of high TGF-β activity and EMT features correlates with poor outcome and metastatic spread [166]. The production of TGF-β by stromal cells appears to be particularly relevant for the development of such phenotypes [167,168]. The relevance of TGF-β signaling in cancer-associated fibroblasts was also proposed to regulate tumor fibrosis and immunity in pancreatic and lung cancer [169,170]. It was also recently shown that TGF-β activated stroma leads to T-cell exclusion from tumor cells (further discussed below) [171,172]. Recent immunogenomic data collected from more than 10,000 human tumors has identified six immune cancer clusters based on several features, including TGF-β gene signatures. TGF-β characterized several categories, with the notable exception of “immunologically quiet” cancers defined by poor T-cell infiltration [163]. Thus, one may speculate that TGF-β is central to the interplay between cancer cells, the stroma, and T cells. The sources of TGF-β within cancer microenvironment are varied. The high concentration of TGF-β in tumor microenvironments attracts and converts fibroblastic, myeloid, and lymphoid cells into immunosuppressive, TGF-β producing cells [11,165,173]. Hence, tumor cells, infiltrating myeloid cells, fibroblasts, and Tregs, secrete TGF-β in several neoplastic conditions, but conventional T cells likely contribute themselves to the TGF-β-rich milieu found in most cancers (Figure 2). Donkor et al. found that TGF-β-deficient T cells were more effective at mediating immune surveillance and curtailing tumor growth in murine systems, thereby unveiling that T cells are a significant source of TGF-β that impede anti-tumor responses [73,174]. Interestingly, in their models of prostate cancer, CD4^+^ T-cell-derived TGF-β (conventional and Tregs) was relevant to the prevention of spontaneous tumor formation, while conventional T-cell TGF-β production impeded the immune-mediated restriction of tumor growth and metastasis. However, the relative contribution of cancer cell/stroma versus T-cell and other immune cell-derived TGF-β in humans remains ill-defined. Nonetheless, TGF-β is an attractive therapeutic target with which to oppose the pro-tumoral effects of this cytokine on both immune and non-immune related processes. As such, TGFbR kinase inhibitors, antibodies (to neutralize active or latent TGF-β), antisense molecules, and genetic engineering approaches are currently investigated in a variety of human cancers [15,165].

Therapeutic opportunities. Irrespective of source, TGF-β will directly suppress T-cell activation and overall favor immunoregulatory differentiation (Figure 1 and Figure 2, and as reviewed above). Within cancer microenvironments, TGF-β will further accentuate the suppression of T cells by inhibiting dendritic cell, macrophage and neutrophil maturation, and polarization as inflammatory cells, thereby limiting antigen presentation and Th1-promoting cytokine secretion [14,175,176]. Non-immune cells will also impact T-cell biology in a TGF-β-dependent manner (Figure 2). Recent data highlight the importance of TGF-β expression within the stromal component of certain urothelial cancer patients treated with atezolizumab, an anti-PD-L1-blocking antibody [172]. The authors found an association between a TGF-β gene expression signature and poor response to treatment in tumors in which T cells are excluded from the tumor parenchyma and retained in the fibroblast and collagen-rich regions around neoplastic cells. Using a mouse model in which tumor T-cell exclusion is observed, the simultaneous blockade of TGF-β and PD-L1 led to improved CD8^+^ T-cell infiltration and better tumor control. The combination of PD-1/PD-L1 axis blockade and TGF-β inhibition has also shown promising results in a model of metastatic colon cancer characterized by low mutational load, T-cell exclusion, and TGF-β signaling in the stroma [171]. In this study, TGF-β signaling blockade rendered previously resistant colon cancer lesions susceptible to PD-1/PD-L1-blockade. However, the precise mechanisms through which TGF-β inhibition/blockade alters T-cell responses to PD-1/PD-L1 blockade in these models remain unclear. One can speculate on the putative role of the stroma or perhaps consider an additive effect, as TGF-β was shown to increase PD-1 expression in cancer-infiltrating mouse T cells [177,178]. This was shown to occur through direct SMAD-dependent mechanisms, as well as indirectly through the SMAD-mediated inhibition of SATB1 expression, a chromatin organizer/transcription factor that was shown to suppress the transcription of the PD-1 gene. These studies add to a growing body of literature supporting the use of TGF-β blockade in combination with other immunomodulatory agents such as OX40 agonistic antibodies [179], IL-2 [180], or even radiation therapy [181]. Pre-clinical evidence also supports the rationale of combining TGF-β and vascular-endothelial growth factor (VEGF) blockade to synergistically enhance anti-tumor immunity [182]. Likewise, the combination of TGF-β with oncolytic viruses can potently increase immune-based tumor control [183]. In addition, the inhibition of TGF-β in combination with vaccination has also shown great promise in pre-clinical settings [184,185,186,187,188] but has not provided a clear indication of clinical efficacy to this date [15]. Likewise, early phase studies using single agents to block TGF-β conversion from latent sources or TGF-β signaling have yielded conflicting results, some of them nonetheless encouraging in subgroups of patients (reviewed in [14,15]). Hence, despite a strong rationale supporting the use of TGF-β blocking agents or small molecule signal inhibitors in order to reverse the multifaceted role of TGF-β within cancer microenvironments, there is currently limited clinical data demonstrating substantial objective cancer responses or evidence of clinically significant immune reactivity against neoplastic cells. However, investment in rationally planned combination trials using potent immunotherapeutic strategies may be the best approach to harness the potential of TGF-β inhibition to enhance anti-cancer responses. One of such combination approach is to design T-cell therapies that combine precise cancer antigen targeting and TGF-β insensitivity through gene engineering on the same T cell. The ex vivo expansion and engineering of T cells allows for the generation of T-cell therapeutic products that can recognize antigens on tumor cells through their natural or artificial receptors (e.g., CAR) [189,190,191,192]. The adoptive transfer of such T cells has yielded spectacular clinical results in selected indications, but overcoming the immunosuppressive cancer microenvironment remains a limitation of these therapies [75]. The engineering of dnTGFβR2-expressing ex vivo expanded T cells, either targeted to the tumor through their natural receptors or CARs, has improved the efficacy of adoptive immunotherapy in numerous pre-clinical models [192,193,194,195,196]. A recently reported clinical study using Epstein-Barr virus (EBV)-specific T cells overexpressing a dnTGFβR2 on eight refractory EBV^+^ Hodgkin’s lymphoma patients showed that the transferred T cells can expand and persist after infusion. These T cells brought partial or complete responses in half of the patients without significant side effects [197]. These encouraging results should nonetheless be confirmed in larger cohorts.

## 6. Conclusions and Perspectives

Most cell types that compose the neoplastic microenvironment are impacted by TGF-β signaling. Although the role of TGF-β on T-cells in the context of cancer has been extensively studied, several uncertainties will require clarification before we will be able to fully harness the therapeutic potential of TGF-β signaling modulation in clinical settings. The current strategies aiming at interrupting TGF-β signaling, either systematically or in T cells alone, will require careful monitoring to define how the negative effects of TGF-β on T cells can be abrogated without compromising homeostatic functions and plausible beneficial effects on certain inflammatory T-cell subsets. Given the widespread autoimmune phenotypes found in mice deficient in TGF-β or components of the TGF-β signaling pathway, careful assessments of toxicities will be required. Although the initial studies using TGF-β inhibition were reassuring from a safety stand point [165], the future of immune-oncology will be based on combination immunotherapies, and whether TGF-β blockade/inhibition will potentialize the immune toxicities of currently used agents such as checkpoint inhibitors is currently unknown [198]. Likewise, systemic TGF-β inhibition may have important adverse effect on tissue homeostasis, including hematopoietic stem cell quiescence.

Thus, one can anticipate that important variables to maximally exploit a yet undetermined therapeutic window will be TGF-β concentration, the timing of the TGF-β signaling blockade, and combinations with other treatments. A study by Nizard et al. illustrates how important the timing and target T-cell population are in the context of TGF-β signaling modulation [199]. In their Human Papilloma Virus (HPV) cancer model, the inhibition of Trm differentiation through TGF-β blockade in the context of vaccination was associated with decreased T-cell tumor infiltration and increased cancer mortality. Hence, mobilizing the TGF-β-mediated effects on Trm differentiation may improve the immune targeting of cancers, but inhibition of TGF-β signaling within tumors can make infiltrating T cells more cytotoxic. The design of molecular switches enabling T cells to benefit from the positive effects of TGF-β on Trm or Th9 differentiation or maintenance of T-cell memory, for instance, while permitting the timely abrogation of TGF-β within tumor microenvironments, may finally offer an opportunity to exploit the full spectrum of TGF-β’s pleiotropy in cancer T-cell therapy.
